# Genome editing and cancer therapy: handling the hypoxia-responsive pathway as a promising strategy

**DOI:** 10.1007/s00018-023-04852-2

**Published:** 2023-07-21

**Authors:** Emanuela Stampone, Debora Bencivenga, Maria Chiara Capellupo, Domenico Roberti, Immacolata Tartaglione, Silverio Perrotta, Fulvio Della Ragione, Adriana Borriello

**Affiliations:** 1 Department of Precision Medicine, University of Campania “L. Vanvitelli”, Via Luigi De Crecchio, 7, 80138 Naples, Italy; 2 Department of the Woman, the Child and of the General and Specialty Surgery, University of Campania “L. Vanvitelli”, Via Luigi De Crecchio, 2, 80138 Naples, Italy

**Keywords:** Von Hippel-Lindau, Hypoxia-inducible factor, PHD, Genome editing, CRISPR/Cas9, Hypoxia sensitive-CAR T

## Abstract

The precise characterization of oxygen-sensing pathways and the identification of pO_2_-regulated gene expression are both issues of critical importance. The O_2_-sensing system plays crucial roles in almost all the pivotal human processes, including the stem cell specification, the growth and development of tissues (such as embryogenesis), the modulation of intermediate metabolism (including the shift of the glucose metabolism from oxidative to anaerobic ATP production and vice versa), and the control of blood pressure. The solid cancer microenvironment is characterized by low oxygen levels and by the consequent activation of the hypoxia response that, in turn, allows a complex adaptive response characterized mainly by neoangiogenesis and metabolic reprogramming. Recently, incredible advances in molecular genetic methodologies allowed the genome editing with high efficiency and, above all, the precise identification of target cells/tissues. These new possibilities and the knowledge of the mechanisms of adaptation to hypoxia suggest the effective development of new therapeutic approaches based on the manipulation, targeting, and exploitation of the oxygen-sensor system molecular mechanisms.

## Introduction

The molecular response to oxygen pressure variation in humans is substantially mediated by activation of the hypoxia-inducible transcription factors (HIF) through heterodimerization of α and β subunits [1, 2]. Low oxygen levels increase HIF-α protein content that translocates into the nucleus, where it interacts with HIF-β protein. The heterodimer recognizes the so-called hypoxia response elements (HREs) generally located at the promoter of target genes. In a strict context-dependent fashion, HIF heterodimers, along with many transcriptional coactivators, modulate the expression of numerous genes, allowing the response to O_2_ pressure (pO_2_) changes at local and systemic levels [1, 3, 4].

While HIF-β protein (also known as arylhydrocarbon receptor nuclear translocator) is constitutively present, HIF-α subunit shows in normoxia a short half-life being rapidly degraded by a RING-E3 ubiquitin ligase/proteasome mechanism [5–9]. The HIF-α ubiquitinating complex includes Cullin 2, Ring Box 1 (RBX1), Elongin C, Elongin B, and the von Hippel Lindau (VHL) tumor suppressor protein [5–9]. VHL, i.e., the substrate recognition subunit of the ubiquitinating complex, recognizes HIF-α by interacting with an unusual degron, which is represented by two hydroxylated prolines (P402 and P564 in human HIF-1α; P405 and P531 in human HIF-2α) located in the so-called oxygen-dependent degradation domains (ODDs) [9, 10].

Three different HIF prolyl 4-hydroxylases (i.e., PHD1-3, prolyl hydroxylase domain-containing enzymes) catalyze the hydroxylation reactions employing oxygen and α-ketoglutarate as substrates. Thus, PHD-dependent HIF-α prolyl hydroxylation should be viewed as a pO_2_ sensor [11, 12]. HIF-α is also hydroxylated on an asparaginyl residue localized in the C-TAD (C-terminal transactivation domain). The reaction is carried out by FIH (factor-inhibiting HIF) and negatively affects the HIF transactivatory function by modifying the recruitment of coactivators [13, 14].

Many HIF target genes are of clinical relevance, especially in relation to cancer and ischaemic diseases. HIF targets include genes encoding erythropoietin (EPO) and vascular endothelial growth factor (VEGF), which induce the production of red blood cells and angiogenesis, respectively, and many other genes involved in metabolic and physiological adaptations to hypoxia [15–17]. Modulation of the HIF system for therapeutic benefit is hence of considerable interest. To date, major efforts have focused on (1) the upregulation of HIF target genes (e.g., EPO) to treat anemia and (2) the inhibition of HIF transcriptional activity as a cancer therapy. However, multiple other therapeutic applications of HIF system targeting can be envisaged, such as wound healing and stroke treatment [18, 19]. Furthermore, because HIF is a pleiotropic transcription factor rapidly and efficiently induced by a small gaseous molecule, it is an attractive model system for basic studies on gene expression control [20–23].

The regulation of protein–protein interactions by oxygen-dependent post-translational modifications is central to the hypoxia-sensing capacity of the HIF system. It has been found that hydroxylation of HIF-α subunits signals their degradation and regulates the transcriptional activity of HIF [10, 24]. Identifying these modifications and the enzymes responsible for them has opened up a new vista in oxygen-dependent signaling, the relevance of which extends far beyond the HIF system. However, other protein–protein and protein–nucleic acid interactions might play central roles in the HIF system and offer therapeutic possibilities. The purpose of this review is to give an overview of the HIF pathway, its cancer alterations, and the potential importance of HIF pathway gene editing in cancer treatment. We briefly overview the HIF system, focusing on the main actors' features of the processes.

## Hypoxia-response pathway

### Von-Hippel Lindau gene and protein

In 1993, the human *VHL* gene was mapped to chromosome 3p25 by genetic linkage studies performed on patients affected by a familial autosomal dominant syndrome characterized by several highly vascularized cancers [25–28]. The syndrome was initially reported in 1904 by the ophthalmologist Engen von Hippel [29] and, subsequently, by Arvid Lindau, who described in 1927 the occurrence of angiomas of the cerebellum and spine [30]. The association of VHL disease to clear cell renal cell carcinoma was reported later in the 1970s [31].

*VHL* gene is formed by three exons. In particular, exon 1 is of 1180 bps and includes a 3′-UTR of 840 bps and a coding sequence of 340 bases. Exon 2 is of 123 bases while exon 3 is of 2434 bps of which 179 bps belong to the ORF. Three different ORFs were identified. One ORF, including exons 1/2/3, is of 642 bps corresponding to a 213 residue protein (pVHL213 or pVHL30) [32–34]. An alternative ORF includes exons 1/3 and corresponds to 172 amino acid proteins (pVHL172 or pVHL17). A third ORF uses a distinct ATG and produces a mature transcript including part of exon 1 (181 bps), exon 2, and exon 3 (pVHL160 or pVHL19) [34]. Recently, additional transcripts have been identified that employed an intronic E1 exon (E1′) of 259 bps starting at 576 bps downstream the 3′-end of intron 1. The use of the novel E1’ exon originates two novel transcripts, including E1/E1′/E2/E3 and E1/E1′/E3, both with a stop codon in the E1’ exon. The translated protein should be of 193 residues, 114 from E1 and 79 from E1’ [35].

The 213 aa VHL protein might be structurally divided into two major domains: an NH2-domain of approximately 100 residues (named β-domain) and a carboxy-terminal domain defined as α-domain. The β-domain and α-domain are held together by two linker sequences (residues 154 to 156 and 189 to 194) and a polar interface [7, 36–38]. β-Domain has been reported to recognize the hydroxylated prolines of the target protein substrates. HIF-1α, considered the main substrate, is hydroxylated on P402 and P564, both placed in ODDs. While the details of hydroxyproline 564 (HyP564) binding to VHL have been extensively clarified, the recognition of HyP402 by VHL is scarcely defined [10, 39, 40]. More intriguing and confirmed by different authors is the observation that hydroxylation reactions occur at different oxygen concentrations [40–42]. It has been demonstrated that the loss of the hydroxyl group on P402 (giving a partial HIF increase) occurs at mild hypoxia. This suggests that the occurrence of two proline hydroxylation sites might represent a mechanism to graduate HIF stability in response to a range of oxygen levels, with P402 playing a minor role and P564 a major role [41]. As a matter of fact, at 10% oxygen (around pO_2_ of 75 mmHg), the substitution of proline 564 with alanine abrogates HIF binding to VHL, while the mutagenesis at P402 scarcely affects the interaction. This suggests that P564 hydroxylation is more dependent on tissue pO_2_ [40–42].

The α domain of VHL (residues from 155 to 192) consists of three α helices (named H1, H2, and H3) that together with one helix of Elongin C forms a structure reminiscent of a four-helix cluster (“folded leaf”) [43–46]. Elongin C was first identified as one of the three subunits (Elongin A, B and C) of the transcription elongation factor required to strongly promote the elongation activity of the RNA polymerase [47]. It was later found that the Elongin BC complex is structurally related to the substrate recognition subunits, acting like scaffold proteins of E3 ubiquitin ligases (as in the VCB-CR complex in association to VHL), not only to target for degradation the RNA polymerase [48, 49]. These findings underlined the importance of VHL as tumor suppressor by modulating transcriptional process. On the other hand, recent findings associate Elongin C somatic mutations with VHL disease [50].

**Fig. 1:**
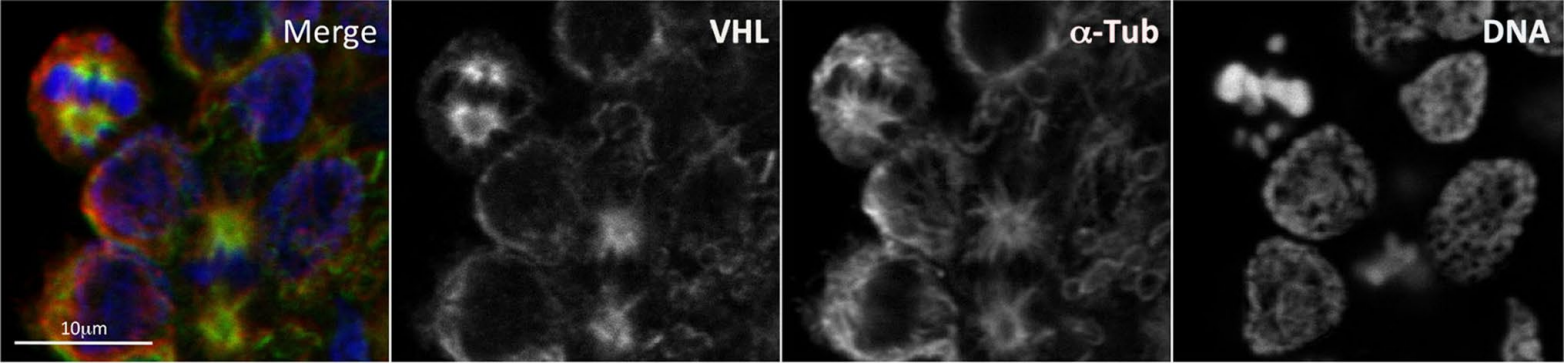
VHL localization on microtubules of the mitotic spindle. Localization of VHL on the mitotic microtubule spindle is investigated by indirect co-immunofluorescence of VHL and α-Tubulin in activated Peripheral Blood Mononuclear Cells (PBMCs). The PBMCs were purified from whole blood of healthy donors using discontinuous density gradients and activated with Phytohaemagglutinin. Hoechst was used for DNA staining. Merge, overlapped images. Scale bar, 10 μm. The immunofluorescence was performed as previously described [230, 231]

In addition to being a key component of a ubiquitin-ligase complex, VHL was reported to localize at mitotic spindle where it exerts a regulatory function promoting a proper spindle orientation [51–55]. Figure 1 shows unpublished confocal data from our laboratory confirming the localization of VHL on the mitotic spindle. Accordingly, VHL alterations might result in spindle misorientation and chromosome instability [51–55]. Additional findings also correlate VHL with primary cilium and suggest that cystic lesions of kidney in subjects with mutated *VHL* gene might be due to primary cilium altered function [56–58]. Since renal cystic lesions represent an early pathological condition that predisposes to tumor progression [56], both VHL role in mitotic spindle and primary cilium might be important in VHL syndrome carcinogenesis.

### Hypoxia-inducible factor genes and proteins

Three HIF-α proteins exist in humans: HIF-1α, HIF-2α, and HIF-3α. While the first two isoforms are largely superimposable functionally and structurally, HIF-3α presents several peculiar features. *HIF1A* gene is localized on chromosome 14q24. It spans about 53 Kb and includes 15 exons [1]. At least 3/4 isoforms were described, of which isoform 1 (826 residues) is probably the most abundant. *HIF2A* (*EPAS1*) gene is localized on chromosome 2p21 and includes 16 exons [59]. The major protein isoform consists of 870 residues. The two HIF-α proteins show different expression patterns and do not regulate the expression of an identical repertoire of target genes [60–62].

**Fig. 2:**
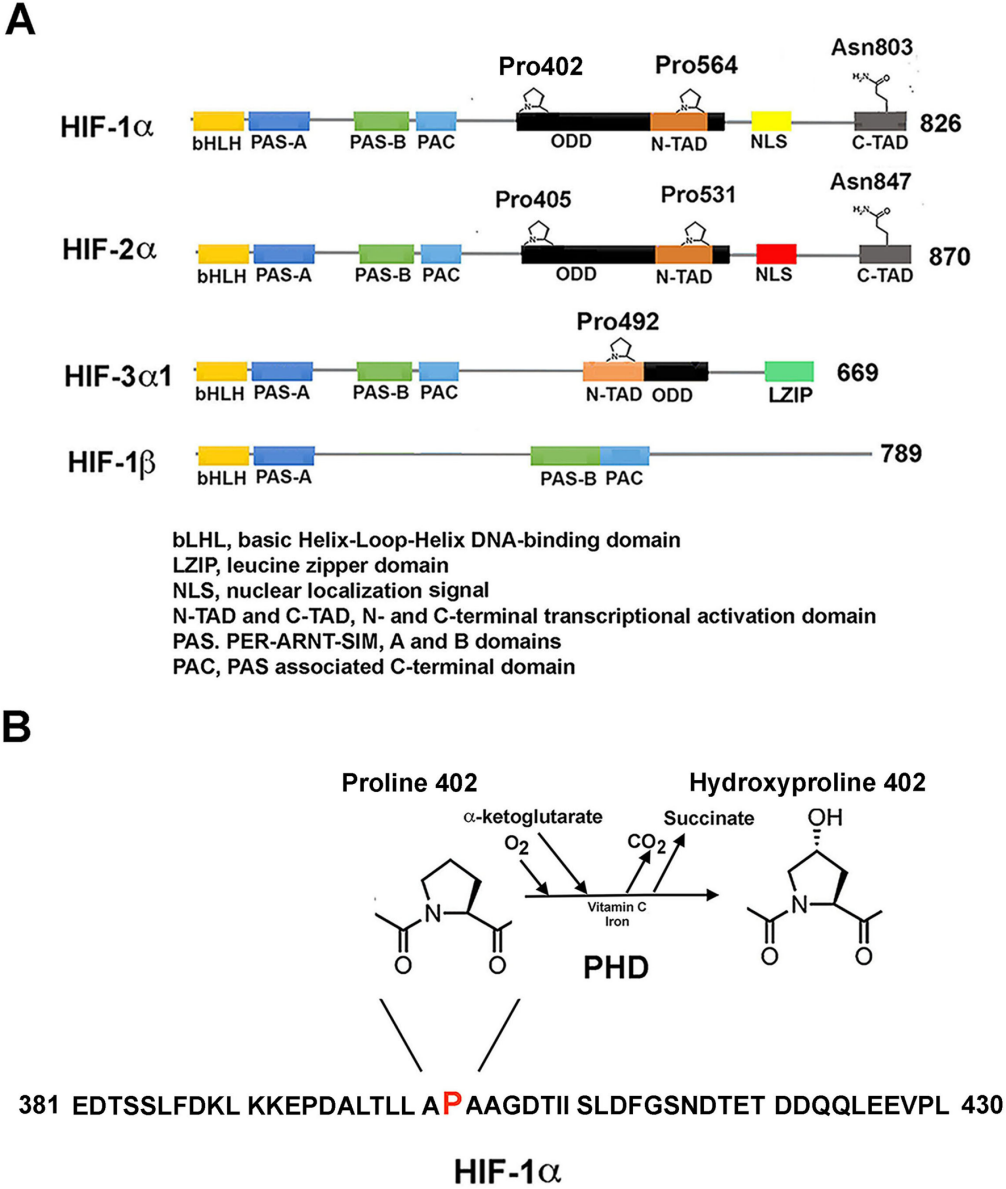
Protein domain arrangement of HIF proteins. **A** Schematic representation of the structural domain organization of HIF proteins belonging to bHLH/PAS protein family of transcriptional regulators: three HIF-α paralogs (HIF-1α, HIF-2α and HIF-3α) and the common interacting partner, HIF-1β (ARNT). Starting from the N-terminus, the following domains are present: bHLH, basic helix–loop–helix DNA-binding domain; PAS, PER-ARNT-SIM, A and B tandem domains, required for protein–protein binding and dimerization; PAC, PAS-associated C-terminal domain. HIF-α paralogs also present an ODD, oxygen-dependent degradation domain; N- and C-term TAD, transactivation domains, involved in the transcriptional activation. Other highlighted motifs are: NLS, nuclear localization sequence; L-ZIP, leucine zipper, DNA-interacting motif present at the C-term of HIF-3α. Specific hydroxylated (Pro402, Pro564, and Asn803 in HIF-1α and Pro405, Pro531, and Asn847 in HIF-2α) and ubiquitinated residues (Ub, Lys 467 in HIF-1α) and the enzymes responsible for the post-translational modification are reported. PHD(1–3), Prolyl Hydroxylase Domain-containing enzyme; VHL, Von Hippel-Lindau protein. **B** Schematic representation of the reaction of hydroxylation on Pro402 of HIF1α by PHD

The domain structure and major functional regions of HIF-1α and HIF-2α are reported in Fig. 2. The sequence of identified domains starts with a b-HLH (basic helix-loop-helix, 17-70/71 aa in HIF-1α) domain followed by two PAS (Per-Arnt-Sim) domains PAS-A and PAS-B, 85–158 and 228–298 aa (HIF-1α) [1]. Then, N-ODD, C-ODD, N-TAD (N-terminal transactivation domain, 531–575 HIF-1α), and C-TAD (786–826 aa HIF-1α) have been described. The complete ODD includes the interval from 401 to 531, while between the two TAD domains, an inhibitory domain (defined as ID) was reported [25]. These intervals must be considered not excessively precise and the activities associated with their functions probably involve different domains. The b-HLH/PAS domains are mostly implicated in heterodimerization and binding to DNA. HIF-1α ODD region includes the two hydroxylatable residues (P402 and P564), while the corresponding residues on HIF-2α are P405 and P531. The two TAD regions are correlated to transcriptional activity. C-TAD is also involved in the binding with several coactivators, including p300 and CBP. The FIH asparagine substrate (N803 in HIF-1α and N851in HIF-2α) is also located in the C-TAD [63, 64]. The mechanisms by which oxygen modulate HIF hydroxylation are described in the next paragraph.

Two aspects of HIF proteins are important. One is that the transcriptional complexes including these proteins are extremely dynamic. The second is that HIF-α level might be regulated not only by the hydroxylation mechanism but also by several transduction pathways and post-translational modifications. However, here, we focus briefly on the organization/modulation of transcriptional complexes that include HIF-α/ARNT heterodimer.

The best known and important interactors of HIF heterodimers are CBP and p300, two protein acetyltransferases that, utilizing their CH1 (cysteine/histidine-rich 1) domain, interact with the HIF-α C-TAD domain. The hydroxylation of the asparaginyl residue localized in C-TAD and catalyzed by FIH hampers the binding. It was also reported that HIF-1α N-TAD recruits CBP by a CH3 domain. An additional protein, CBP/p300 interacting ED-rich tail 2 domain (CITED2), blocks the interaction of HIF with CBP by competing with the CH1 domain [65–67].

Although CBP and p300 are frequently cited together for their high homologies, many studies demonstrated that they participate in and modulate more than 400 transcriptional complexes. In addition, and more importantly, the HIF/ARNT transcriptosome complex, including CBP, might regulate an array of genes partially distinct from those controlled by HIF/ARNT bound to p300. CBP/p300 modulates transcription by acetylating lysyl residues on histones and non-histone proteins, such as transcription factors or transcriptional co-regulators. The acetylation should enhance/permit the expression of target genes. Specifically, P300 was reported to acetylate HIF-α on K709, increasing its stability and, thus, the transcriptional activity of the heterodimer. Conversely, CBP (not p300) acetylates HIF-2α (modulating EPO expression, among others). In this case, the interaction does not necessarily involve C-TAD. Thus, p300 controls HIF-1α and CBP HIF-2α [68, 69]. Additional proteins modulate the interaction of CBP/p300 with the HIF-1(2)α/ARNT heterodimer, either positively or negatively. For instance, PKM2 and Src1 proteins facilitate this interaction while FHL (four and a half LIM domain) protein negatively affects the CBP/P300 binding with the HIF heterodimer [70, 71].

Other proteins alter the formation of HIF-α/ARNT complex. For example, FBP1 (Fructose 1,6-bisphosphatase), COMMD1 (COMM domain-containing 1), testis-specific gene antigen 10 (TSGA10), and  MgcRasGAP (male germ cell Rac GTPase activator protein) negatively regulate the heterodimerization process [72–74].

The human *HIF3A* gene is located on chromosome 19q13.2 spanning 43 kb and contains 19 exons. More than ten different human HIF-3α transcripts were reported due to alternative splicing. These variants are expressed at different times of development and in different tissues. The largest protein isoform includes 669 residues (HIF-3α). Compared to HIF-1α and HIF-2α, the HIF-3α domain sequence lacks the C-TAD domain (and thus the asparaginyl hydroxylation by FIH), which is substituted by the L-ZIP (leucine-zipper) domain. Probably, the protein presents only one hydroxylatable proline (P490) involved in the pVHL binding. Intriguingly, the ODD domain is absent from several HIF-3α variants [75–77]. These isoforms are unlikely to be regulated by PHD/VHL-mediated degradation. Thus, only some of them are regulated by hypoxia, whereas other isoforms are regulated by signals other than O_2_ pressure. HIF-3α functions are complex, and till now, few were known. Several aspects have emerged in recent years. Particularly, some isoforms frequently different cellular localization with opposite functions. For example, HIF-3α4 forms dimers with HIF-1α, acting as a dominant-negative, lacking the L-ZIP and ODD domains [78]. Moreover, some isoforms show a transcriptional activity independent of oxygen pressure, and bind to response elements different from HREs [79].

### Prolyl hydroxylase genes and proteins

*PHD1* gene is localized on chromosome 19q13.2, includes six exons, and encodes a protein of 407 residues. *PHD2* gene is localized on 1q42.2 chromosome, comprises five exons, produces a protein of 426 amino acids. The third *PHD* (*PHD3*) maps on 14q13.1, and includes six exons [80, 81]. Mature PHD3 protein is of 239 residues [80, 81]. PHD2 is constitutively expressed and is the most abundant isoform, except in the testis and heart. The protein comprises a long N-terminal intrinsically unstructured region (residues 1–187) and a well-structured oxygenase domain representing the actual catalytic center (residues 188–418). This region involves the sequence that recognizes HIF protein, the so-called “facial triad” (H313, D315, H374) that binds ferrous ions, and the residue interacting with α-ketoglutarate (R383). PHD2 (as the other two PHDs) is functionally an oxidoreductase enzyme that incorporates both atoms of a molecule of oxygen into substrates [81]. In other words, these enzymes are dioxygenases, contraposes to monooxygenase.

Detailed kinetic studies suggest that C-ODD is a better substrate than N-ODD [82, 83]. The difference is due mainly to the efficiency of releasing the hydroxylated peptide. More interesting is the comparison between the affinity (Km) for oxygen measured at saturating conditions of the other substrates. The data obtained (albeit not conclusive) allow the conclusions that oxygen is not bound very tightly and that the Km is higher than oxygen concentrations. This indicates that oxygen is rate limiting and that these dioxygenases might represent good oxygen-sensors. PHD-2 is mostly cytoplasmic, but its nuclear localization appears massive during cancer progression [82, 83]. A zf-MYND domain was evidenced (residues 21–58) at the PHD-2 N-terminus. zf-MYND might act as a PHD-2 inhibitor [84], thus suggesting the existence of a specific regulative pathway for this enzyme. zf-MYND binds several regulatory proteins (FKBP 38 and SPOP) that can mediate the proteasomal degradation of PHD-2. Several PHD2 putative phosphorylation sites were also identified, suggesting that the enzyme is regulated by this post-translational modification.

*PHD1* is highly expressed in the testis, while *PHD3* is mostly expressed in the heart. The ablation of the three PHD genes results in distinct phenotypes. *Phd2*-deficient mice show embryonic lethality due to placental and heart alterations [85]. Inactivation of the gene in the postnatal period results in polycythemia, augmented angiogenesis and heart failure. Loss of PHD1 might cause the reprogramming of energy metabolism and improved tolerance to hypoxia. Finally, the inactivation of PHD3 determines diminished blood pressure and adrenal system hypofunction. The expression of PHDs is regulated at transcriptional and translational levels [86]. At the level of protein stability, the prolyl cis/trans isomerase FK506 binding protein 38 (FKBP38) was identified as a negative regulator of PHD2 [85], whereas the ubiquitin ligase SIAH2 (seven in absentia homolog 2) was shown to mediate the degradation of PHD3 [87].

## Genetic changes in the hypoxia-response pathway and human cancer

The correlation between genetic alterations in the hypoxia-response pathway genetic alterations and the development and progression of cancer was demonstrated in both hereditary conditions and sporadic tumors. For clarity, we initially describe cancers occurring in the VHL syndrome and, then, sporadic cancers with mutations in *VHL*, *HIF,* and *PHD* genes.

### VHL syndrome

VHL disease is due to heterozygous germline mutations acting through an autosomal dominant and almost complete mechanism (97%) [88]. The disease is generally classified into two major types, i.e., type 1 and type 2. Type 2 VHL syndrome might generally present RCC (renal cell carcinoma), PCC (pheochromocytoma/paraganglioma cancer), retinal hemangioblastomas, tumors of CNS (central nervous system), pancreatic NET (neuroendocrine tumors), pancreas and kidney cysts. Type 2 can be classified into 2A (without RCC), 2B (with RCC), and 2C (isolated PCC) [88]. Type 1 VHL disease is characterized by a low risk of developing PCC [88].

Many different *VHL* mutations (more than 1000) have been identified, primarly missense mutations (about 50%), frameshift mutations (13%), nonsense mutations (11%), large deletions (11%), splicing mutations (7%) inframe deletions/insertions [89]. The most common germline genetic changes include delF76, N78S, Rl61Stop, R167Q, Rl67W, and L178P [89]. It is noteworthy that type 2 VHL disease mostly shows missense mutations (from 85 to 92%) that particularly interest codons 167 and 238. Type 1 VHL disease is characterized by truncating alterations. In contrast, somatic *VHL* genetic changes were identified in a large percentage (from 50 to 70%) [89].

The heterogeneity of mutations is the probable cause of the distinct phenotype observed. Similarly, a different genetic background might explain why the same mutation might result in distinct phenotypes. Additional studies allowed a different stratification of *VHL* missense mutations. Missense mutations involving the binding site with HIF-α or not involving this binding site were differentiated [90, 91]. It is also noteworthy that VHL exerts function unrelated to the control of HIF level/activity (like the above described control of mitotic spindle and primary cilium organization) and thus independent of pO_2_. These activities might be also associated with PCCs development. Unexpectedly, the VHL type 2C phenotype might increase HIF-α degradation.

VHL disease might be due to a different combination of two molecular hits. In addition to mutations, *VHL* gene expression could be modulated by epigenetic changes. Accordingly, alterations of the epigenetic pattern appear to be responsible for a not yet defined percentage of clear cell RCC. Finally, it is also remarkable that some homozygous/double heterozygous missense *VHL* germline mutations could develop hereditary erytrocytosis. The best known (but not the only one) mutation is c.598C > T (p.R200W) that, in homozygosity, is the etiology of the so-called Chuvash polycythemia [92–96].

### Sporadic cancer with *VHL* mutations

The most common subtype of kidney cancer is the clear cell renal cell cancer (ccRCC) type, representing approximately 75% of cases. *VHL* inactivation was demonstrated in the majority of tumors (50–80% of cases, depending on the study) [97]. Moreover, *VHL* mutation appears to be an early event in cancerogenesis. Prevalent alterations appear to affect codons 65, 114, 147, and 155 [98]. On the other hand, major changes also appear to be distributed in additional codons in exons 1 and 2. Nickerson et al*.* reported an overall frequency of mutation of 85%, with relative percentages of deletion (34%), insertion (24%), missense (24%), nonsense (10%), and splicing (9%). The double mutation was evidenced in 3.4% of cases [99, 100].

### *PHD* mutations and cancer

Heterozygous germline mutations in *PHD2* gene were first reported in familial erythrocytosis [101, 102]. Particularly, it was reported a heterozygous loss-of-function mutation of *PHD2* (c.1121A > G, p.H374R) with the development of both erythrocytosis and recurrent paraganglioma. Functional analysis indicates that H374 is important in the binding of cofactor Fe^2+^, and mutation of this residue is expected to impair the catalytic function of PHDs [101, 102]. Yang et al*.* reported heterozygous germline mutations in PHD1 (c.188T > A, p.S61R and c.682G > T, p.A228S) in patients with polycythemia and PCCs, respectively [103]. Further research detected *PHD2* mutations in non-small cell lung cancer and clear cell ovarian cancers [104, 105]. These data collectively suggest that mutant PHDs are associated with PCCs susceptibility. However, compared to *VHL* and *HIF-2A*, *PHDs* mutations are relatively rare in patients with PCCs.

### *HIF* genes mutations and tumors

The *HIFA* genes family includes three members: *HIF1A*, *HIF2A*, and *HIF3A*. *HIF1A* presents few mutations when matched to *HIF2A* [106, 107]. Specifically, the ClinVar database (https://www.ncbi.nlm.nih.gov/clinvar/) presents only 33 records related to this gene. A germline missense change (p.A475S) and a somatic mutation (p.V116G) were described in a case of RCC associated with *VHL* mutation. However, it is important to note that p.A475S is probably a benign change. On the other hand, several *HIF1A* SNPs were demonstrated to be associated with human cancers [106, 107]. *HIF2A* is frequently mutated in hereditary erythrocytosis associated or not to PCC. Some germline *HIF2A* mutations (p.M535V, p.G537R, p.G537W) only result in polycythemia but not in cancers, suggesting that *HIF2A* mutation is not sufficient for cancer development [108]. Literature data show that p.G537R change might give erythrocytosis and needs *VHL* mutations to originate an RCC. Similarly, other mutations on the exon 2 of *HIF2A* (i.e., p.F374Y) and exon 9 (p.F374Y) cause erythrocytosis and predispose to PPC. Also, *HIF2A* exon 12 somatic changes (p.A530T and p.A530V) give origin to PCCs [109]. These data allowed the hypothesis that residue alteration near P531 (the hydroxylation residue) affects the protein's capability to act as PHD substrate. Two additional *HIF2A* somatic mutations (p.L529P and p.Y532C) were identified in subjects with congenital erythrocytosis, PCCs, and increased levels of serum somatostatin. Other reported *HIF2A* mutations include p.S71Y, p.A530V, p.L529P, and p.L542P in polycythemic patients affected by PCCs.

## Genome editing strategies and human cancers

### ZNF, TALEN, and CRISPR/Cas9 as powerful tools for genome editing

Developing methodologies to target and modify specific genome sequences represents a major breakthrough in recent years. In particular, the availability of distinct nucleases (ZFN, zinc finger nuclease; TALEN, transcription activator-type effector nuclease, and Cas9, a bacterial nuclease directed by an RNA guide) enabled the generation of double-strand breaks (DSB) in select genomic regions [110–112]. The nucleases present distinct mechanisms for identifying the target phosphodiester bond, namely a DNA recognition protein domain (ZFN and TALEN) or an RNA guide (CRISPR/Cas9). Following the nuclease activity, the non-homologous end joining (NHEJ) pathway reconnects the two DNA ends. Since NHEJ is not perfect, it might cause the introduction or removal of sequences creating indels that could alter the open reading frame, causing the target gene to lose activity. Alternatively, a sequence of DNA could be inserted. This strategy might be used to repair negative mutations or, in general, allow genome editing.

A central issue of the method (based on nuclease activity) is the precise identification of the sequence to edit. For this purpose, both ZFN and TALEN employ a protein domain (zinc fingers or sequences similar to transcription activators) that need to be designed, synthesized, and validated [110, 111]. Conversely, CRISPR/Cas9 employs an RNA sequence as a guide (gRNA) that is very easy to design/validate in silico [112]. However, the occurrence of numerous off-targets, a low efficiency of DSBs formation and complex cell/tissue delivering strategies still represent major problems [113–115]. Although it is a complex approach, TALEN has recently been reported more efficient than CRISPR/Cas9 in genome editing, at least for applications in heterochromatin [116]. Additional methodologies subsequently evolved from CRISPR/Cas9, allowing a single base editing (BE) without the necessity of causing DSBs.

### Base editing

BE must be considered an extraordinary evolution of CRISPR/Cas9 in the world of human genome editing. Various improvements significantly ameliorated BE's strategies, and new ones will certainly develop soon. Three major aspects of base editing are briefly discussed: (1) basic BE methods, (2) identification of the base to be edited, and (3) targetability of BE machinery.

**Fig. 3:**
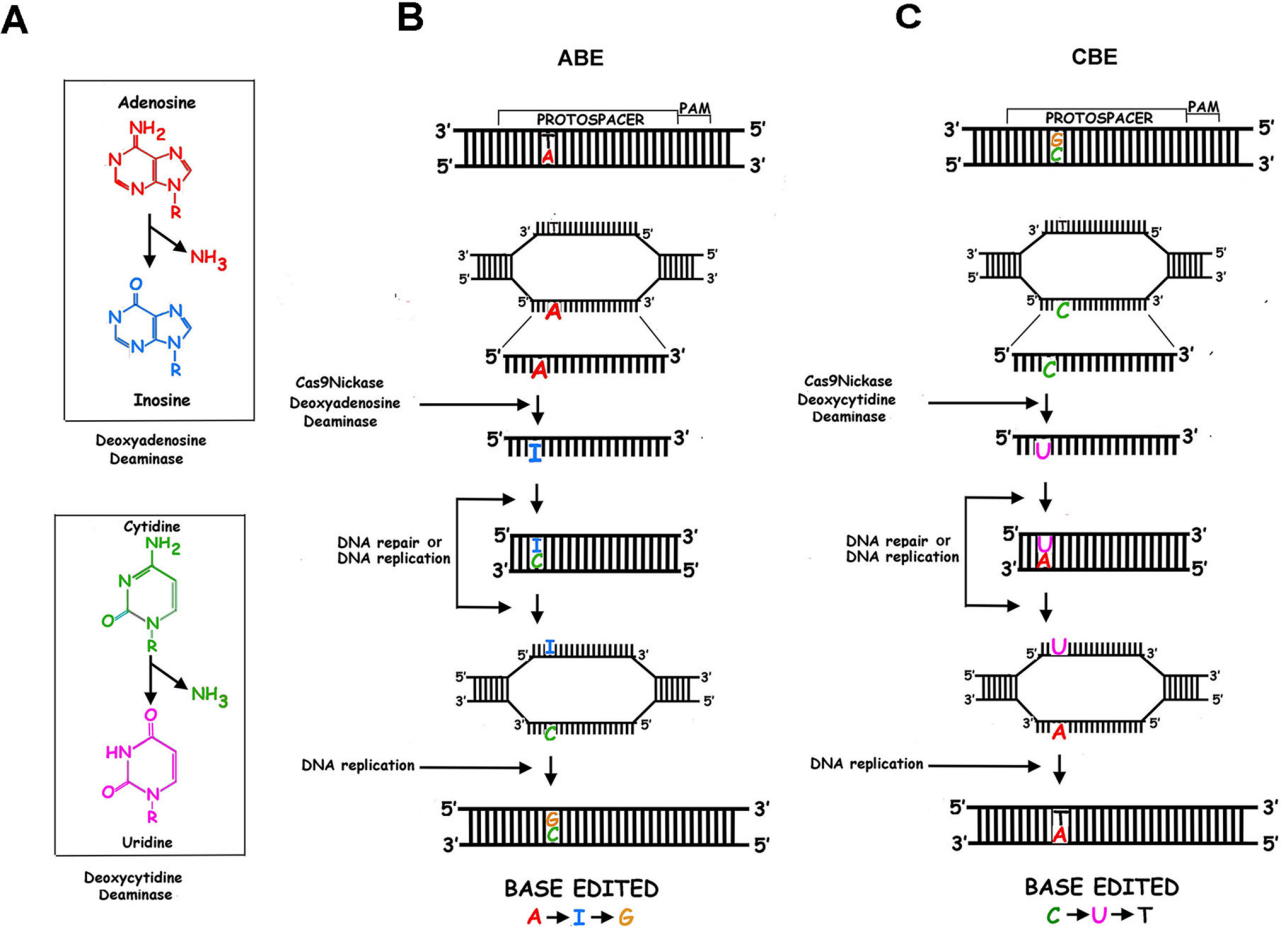
CRISPR-Cas9 Base editing systems. Schematic representation of CRISPR-Cas9 base editing. Cytidine (CBE) and adenosine (ABE) base editing systems both employ a Cas9 nickase (or a catalytically inactive (dead) Cas9, dCas9) respectively fused to a cytidine and adenosine deaminase. In panel **A** are summarized the reactions catalyzed by the two deaminases. (Panel **B**) ABE determines the change of an adenosine (A) into a guanosine (G), while CBE (Panel **C**) mediates the conversion of a cytidine (C) into a thymidine (T)

BE consists of changing cytosine into thymine (CBE) or adenine into guanine (ABE) (Fig. 3). Both changes initially require the deamination of cytosine into uracil or adenine into hypoxanthine and are due to the activity of cytidine deaminase or adenine deaminase capable of acting on DNA [117, 118]. The first version of CBE (i.e., CBE1) includes rat deaminase rAPOBEC1 (i.e., apolipoprotein B mRNA editing enzyme, catalytic polypeptide 1) fused with inactive Streptococcus pyogenes (Sp) dCas9 (dead Cas9). rAPOBEC1 was selected for its high deaminase activity. Inactive dCas9 was unable to create DSB while it could still bind DNA [117]. This first version of BE1 converts a specifically targeted cytosine into uracil. Then, this uracil is identified as a mismatch by the DNA repair engine that usually removes uracil. To hamper uracil removal, in the second version of CBE (i.e., CBE2), uracil glycosylase inhibitor (UGI, a small protein of bacteriophage PBS) was added to the complex [117, 119].

In a subsequent modification of CBE, CBE3, dCas9 (SpCas9n, where n is for nickase, APOBEC1–SpCas9n–UGI fusion) was modified in that the protein was able to remove the G residue (of the couple U:G) occurring in the non-edited DNA strand [120, 121]. The nicking event favors the substitution of G with A (by the repair machinery). Finally, uracil is substituted by thymine by the same repairing engine, forming the desired T:A couple. In the fourth generation of CBE (CBE4), a second uracil glycosylase inhibitor was added, ameliorating editing efficiency and product purity [122, 123]. Alternative cytidine deaminases were also identified for their increased capability of deaminating the pyrimidine base, raising additional complexes for efficient CBE [124, 125].

For ABE development, a dimeric tRNA adenine deaminase from *E. coli* (defined as TadA) was subjected to complex mutagenesis to obtain an enzyme able of deaminating adenine in single-strand DNA. As in CBE3, TadA was fused to SpCas9n [118]. Since TadA works as a homodimer, one of the two subunits was left unmodified (and, thus, active) while the other was inactivated by mutagenesis. Although not the focus of this review, it must be stressed that important studies identified a large series of deaminase mutants with distinct and increasing catalytic capability [126, 127].

A major problem in BE is the precise identification of the cytosine or adenine to be deaminated. The specificity depends on the so-called protospacer adjacent motif (PAM) that dictates the distances between PAM and the substrate [128, 129]. The classical PAM sequence was NGG, where N is an undefined nucleotide. Numerous SpCas9n mutants were generated (that differed in the PAM sequence), thus expanding the targeting base. Moreover, during ABE evolution, seven generations of ABE were created with a base pair efficiency of 50%, remarkable purity (about 99%), and a low rate of indels (no more than 0.1%) [130, 131].

### Prime editing

In 2019, another CRISPR-based genome editing strategy was developed, i.e., the prime editor (PE). In this technique, a Cas9n was fused with reverse transcriptase (RT) and not with a deaminase [132]. Accordingly, the methodology does not employ a guide but a primer template for RT to re-write all the base conversion. The technique has already been experimented in several eye, liver, skin inherited human diseases, neurodegenerative diseases, cystic fibrosis, β-talassemia [133]. PE was also used in vivo for HIF-2α in mouse endothelial cells to inhibit pulmonary hypertension [133]. The possibility to precisely correct point mutations, when the ABE and CBE systems are not applicable, without creating double strand breaks, has been readily exploited in the oncological field to correct missense mutations in undruggable driver genes, such as *KRAS*, *APC,* and *TP53* [135–138]. However, although extremely promising, the method is under development and needs to be improved [139].

### The targetability of base editing machinery

A key issue in genomic editing is how to deliver CRISPR/Cas9 or BE machinery to target cells/tissues with high efficiency and selectivity. This issue’s relevance prompted many studies and different delivery solutions. In brief, DNA or mRNA encoding for genome handling machinery was employed, and viruses or lipid nanoparticles were selected as their molecular carriers. The use of plasmids might represent the simplest way, and several experiments confirmed this view [140]. In contrast, plasmid transfection shows some limitations due to the scarce percentage of transfected cells and toxicity, particularly (but not exclusively) evident in primary cells [141].

Alternatively, viruses (adenoviruses, lentiviruses, and others) could be employed. However, immune response and off-targets were detected in the case of genome editing of hematopoietic cells by lentiviruses [142, 143]. Adenovirus (i.e., adeno-associated viral vectors, AAV) use also showed negative aspects, including a strong immune response, liver damage, and poor cell-specific targeting. The excessive immune response (directed against the transfected cells) is facilitated by a prolonged Cas9 expression and the frequent occurrence of anti-Cas9 antibodies in human sera. AAVs were, however, employed in several experiments, although they have a limited packaging capacity [144, 145].

The transfection of modified/stabilized mRNA encoding for CRISPR/Cas9 or selected mutants of CBE or ABE engines along with gRNA might represent a good alternative to viruses [144, 145]. The methodology was employed in viral vaccines, protein replacement, and cell phenotype modification [144–150].

Several improvements were recently described that appear safe, effective, and showed great potential in therapy. The success of coronavirus vaccines confirmed the relevance of the mRNA strategy and validated its therapeutic use [151]. Lipid nanoparticles were also used for delivery, and numerous chemical structures were developed (described accurately in recent reviews) [152, 153]. Lipid nanoparticles might be injected in the proximity of target cells. However, recent studies also suggested that the targets’ specificity might be increased by inserting, at the surface of lipid nanoparticles, antibodies, or, in general, ligands that can direct the lipidic vectors toward the selected cell phenotype.

### Genome editing and human diseases

The focus of this paragraph is only on studies that, in our view, have clear connections to human diseases. For more thorough data descriptions in the field, we redirect to detailed reviews [154, 155].

Genome editing might either correct disease-causing mutations or eliminate disease-responsible genes. An example is represented by the CRISPR/Cas9-mediated gene editing of *Vegfr2* to abrogate angiogenesis in vivo, testing its effectiveness in two mouse models of laser-induced choroid neovascularization and oxygen-induced retinopathy [134]. Although double-strand nuclease can be employed for both these aims, specific base deamination (i.e., CBE and ABE) seems most appealing (see the above description). On the other hand, the only reported study in humans employed a CRISPR/Cas9 nuclease and a modified RNA guide delivered by lipid nanoparticles [156]. This clinical investigation, published in August 2021, showed the inactivation of transthyretin gene by in vivo editing and consequent correction of transthyretin amyloidosis [156]. The employed gene editing methodology is based on DSBs formation, although, according to a generally accepted view, it is preferable to avoid these DNA modifications and alternatively employ base deaminase as a tool.

Most studies were performed in cellular or mouse models of diseases, while studies in primates that employ CBE/ABE are still rare. Thus, the translation into clinical settings is substantially elusive. The clinical trials under development generally involve the ex vivo handling of hematopoietic stem cells to treat various clinical conditions (thalassemia, sickle cell anemia, X-linked chronic granulomatous disease, severe congenital neutropenia, X-linked hyper-IgM syndrome, SCID-X1, mucopolysaccharidosis type I, and Wiskott–Aldrich syndrome) [157–164].

Several genetic pathologies have been corrected in mouse models and the relative approaches are still in a preclinical status [165–179]. Conversely, two intriguing base editing studies have been performed in primates (macaques and cynomolgus monkeys) to inactivate the *PCSK9* gene and reduce low-density lipoprotein and hematic cholesterol levels. Importantly, lipid nanoparticles were employed for the delivery [180, 181]. In the cynomolgus monkey report, the ABE approach (using ABE 8.8 mRNA) was used to alter splice donor at the boundary of *PCSK9* exon 1 and intron 1 with an efficiency of about 50% editing in the liver [182]. A similar methodology was used in the macaques' study (i.e., lipid nanoparticles, ABE mRNA, and a splicing acceptor as target) [181]. However, about 26% editing was demonstrated in this study [181].

Investigations into the use of genome editing in cancer treatment are so far much more limited than in genetic diseases therapy. As a result, there is scarce conclusive data from clinical trials [182]. Indeed, there are limits not yet overcome and on which scientific efforts have focused in recent years, such as improvement of delivery methods, off- targeting, the unsolicited generation of mutations in the *p53* gene, the immunogenicity of CRISPR-Cas9 systems and, not less important, the ethical requirement. In other words, efficacy and safety have to be improved for a proper design of a clinical trial. Thus, untill now, gene editing for cancer treatment has been an appealing and promising technology, but still at pre-clinical stage, at least for solid tumors.

We focus our review on specific aspects of this topic, particularly on ex vivo*/*in vivo DNA editing in adoptive cell therapies (including CAR-T cells, chimeric antigen receptor T cells) and ex vivo*/*in vivo silencing of a dominant oncogene. Although the possibility of correcting an inactivating mutation of tumor suppressor genes is a fascinating possibility, very few data exist in the literature on this topic.

Adoptive cell therapies (ACTs) include the isolation/expansion of endogenous T cells against tumor antigens, the introduction of transgenic T cell receptors to redirect their specificity and transduction in T cells of the chimeric antigen receptor (CAR) [183]. In particular, CAR-T cells strategy requires preparation of T cells from the patient, introduction (by a virus) of CAR to develop a specific cytotoxic activity, and, finally, a re-introduction in the human body of the modified cells. Although CAR-T approach produced remarkable antitumoral clinical responses, some limitations were evident [184]. These include a limited trafficking of cells, the possibility of antigen escape, and scarce tumor infiltration. In addition, the local tumor environment alters/reduces the T cell-dependent activity. Different genome editing strategies have been proposed to overcome these negative effects and enhance CAR-T anticancer activity. In particular, almost all the approaches include CRISPR/Cas9-dependent inactivation of the gene encoding programmed cell death protein 1 (i.e., PD-1) and, in some instances, silencing the expression of endogenous T cell receptor (TCR) chains, TCRα (TRAC), and TCRβ (TRBC) to increase the activity of the chimeric antigen receptor [185]. In a previous study, Cas9 was delivered by engineered lentiviruses [186]. An alternative ex vivo approach was employed in a recent report. PD-1 was inactivated by delivering Cas9 machinery into T cells by electroporation, and the modified cells were subsequently infused into refractory non-small cell lung tumor patients [186].

A different one-step strategy was recently developed for facilitating the treatment of solid cancers. Lentivirus particles charged with Cas9 ribonucleoproteins and CAR transgene were used to target primary T cells [187, 188]. By using this approach, the specificity of targets (by lentivirus particles) is associated with transient activity (to avoid prolonged genome editing). CAR transgene should allow the expression of a cancer-directed TCR. Other technologies were proposed for specific targeting, including decorating nanoparticles or a method defined by the authors as SORT (Selective ORgan Targeting), wherein different classes of lipid nanoparticles are modified to identify target extra-hepatic tissues [189].

Several studies described the effects of CRISPR/Cas9-mediated inactivation of dominant oncogenes in established human cancer cells. These investigations, based on in vitro experiments and in vivo implantation of modified cells, confirmed the central role of numerous oncogenes in the malignant phenotype. However, these studies are mostly proofs of concept and do not consider human cancer's complex aggressiveness, heterogeneity, and continuous genetic/molecular evolution. An interesting attempt has been to modify the genome of an experimental tumor by CRISPR/Cas directly delivered to animal models. So far, few examples are available. In 2017, co-delivery of Cas9 and a gRNA targeting mutated malignant EGFR (L858R) resulted in reducing tumor size in a mouse model of human lung cancer. Adenovirus was employed as a vector. The virus was intratumorally injected when the implanted cancer reached a sufficient size [190]. Cas9 reduced tumor size by about 80% with few variations considering saline or Cas9 alone (i.e., without gRNA) as controls [190].

Most recently, a single intracerebral injection of CRISPR/Cas9 directed against *PLK1* into an aggressive glioblastoma in a mouse model causes a 70% editing. In this experiment, Cas9 mRNA and sgRNA were encapsulated in lipid nanoparticles formed by a novel amino-ionizable lipid [191]. The treatment induced apoptosis and reduced tumor growth by 50%, thus increasing survival by about 30%. The authors also investigated the possibility of targeting solid metastasizing malignancies in vivo. In this case, they used a disseminated ovarian tumor in a mouse model. Lipid nanoparticles coated with anti-EGFR antibodies were employed for delivering RNA with an efficiency of *PLK1* editing of about 80% [191]. The treatment resulted in about an 80% survival increase. In conclusion, data emerging from genetic disease correction and cancer treatment strongly suggest that genome editing might represent an important strategy to be pursued in the field of precision medicine therapy.

## Genome editing and hypoxia-response pathway

### Strategies targeting hypoxia-related genes for cancer therapy

Hypoxia is a key feature of human solid cancers. Accordingly, tumor cells adapt to the hypoxic microenvironment by increasing HIF-α levels, which in turn, represents an additional factor facilitating tumor development and diffusion. In particular, the hypoxic condition disadvantages the lymphocytes' activity by reducing the local immune response against the malignancy [192, 193]. A HIF-α increase also causes the expression of PD-L1 (PD-1 ligand 1), which further decreases anticancer immunity [194, 195]. HIF-α accumulation induces VEGF production, stimulating angiogenesis and, in turn, facilitating cancer development. In addition, hypoxia facilitates the EMT (epithelial-mesenchymal transformation) process that causes the release of malignant cells from the cancer mass, allowing metastasization that represents a major cause of cancer-dependent death [196]. Although the molecular details of metastasization event are still elusive, the relevance of hypoxia and, in particular, the role of HIF-α (and its targets, including TGF-β, SMAD, TWIST, Snail, Slug, SIP1, and ZEB1) has been confirmed in this process. To be noted, HIF1-α induces the expression of drug efflux proteins, such as MDR-1 (multidrug resistance 1 protein) and MRP-1 (multidrug resistance-associated Protein 1), which belong to the ABCC ATP-dependent transporter family, responsible for the efflux of conventional chemotherapeutic agents used for the treatment of several solid tumors [197]. Finally, metabolic reprogramming and the protection from apoptosis and from DNA damage of cancer cells promoted by HIF-α expression have been associated with cancer therapy resistance [198]. Therefore, all these aspects that contribute to the adaptation of cancer cells to hypoxia suggested that HIF-α targeting could represent a good anticancer strategy. In agreement with these observations, many published and ongoing trials suggest using HIF inhibitors in cancer therapy [199–201]. So far, several classes of HIF inhibitors have been identified, including inhibitors of HIF transcription, translation, and protein stability (including EZN-2208, PX-478, BIX01294, and bortezomib) and inhibitors of HIF heterodimerization (PT2385, PT2399, PT2977, and 0X3) [178, 179]. Several tumors were reported as susceptible to HIF inhibitors treatment, including glioblastoma, pancreatic, breast, and renal clear cell cancers [202–206].

PT2385 is the first-in-class HIF-2α inhibitor that hampers ARNT heterodimerization [203]. This is due to the drug's ability to interact with a cavity within the HIF-2α PAS-B domain. A phase 1 dose escalation/expansion trial demonstrated that the molecule was well tolerated. PT-2385 also showed inhibitory activity against glioblastoma by increasing the survival in orthotopic models of this cancer [206]. The target specificity was confirmed by a more recent study [207].

Very recently (August 2021), FDA approved the use of PT2977 (MK-6482, belzutifan) for cancers associated with VHL disease. Belzutifan was investigated in phase 2, open-labeling, single-group trial of 61 subjects with renal cell carcinoma associated with VHL disease. The study concluded that the molecule showed positive activity in a significant percentage of VHL disease patients with renal cell carcinoma and non-renal cell carcinomas [207]. Belzutifan was also demonstrated efficacious in treating Pacak–Zhuang syndrome, a disease characterized by polycythemia and multiple parangliomas [208] and due to activating mutations of *HIF2A* gene [209].

*HIF2A* gene silencing was also obtained using short interfering RNA. Data in the literature demonstrated that this treatment could counteract glioblastoma cell phenotype [210].

Altogether, these data strongly suggest that a selective genome editing of *HIF1A/HIF2A* gene might represent a more stable strategy for HIF down-regulation. This is particularly relevant for difficult-to-treat tumors like brain cancer and pancreatic carcinoma. Unfortunately, scarce data exist to confirm this view experimentally. HEP-2 cells CRISPR/Cas9 deleted of HIF-1α (and its target Glut-1) were prepared [211]. These cells showed an apparent decrease in proliferation, migration, and invasiveness. An additional intriguing finding regards transarterial embolization, a palliative strategy employed in non-resectable hepatocellular carcinoma [212]. The strategy frequently fails since HIF-1α induces VEGF and other factors facilitating the development of new vases. A subcutaneous hepatic cancer was injected with a lentivirus delivering CRISPR/Cas9 system targeting *HIF1A*, and a remarkably low level of HIF-1α was verified. Accordingly, a significant reduction of microvascular density and cancer development was evidenced by increased animal survival [212].

### Strategies to exploit hypoxia for cancer therapy

The main objectives currently achievable for genomic editing in cancer therapy might be summarized as: (a) engineering CAR T cells for ameliorating their efficiency, and (b) silencing of specific (onco)genes for facilitating/inducing tumor cell death. Major obstacles in applying these therapeutic strategies include the difficulty of targeting a specific malignant phenotype, the still limited editing efficiency, and the occurrence in human serum of antiCas9 antibodies. However, two major strategies are under implementation that use hypoxia-related molecular mechanisms as a selection criterion for a more effective and less toxic cancer therapy.

**Fig. 4:**
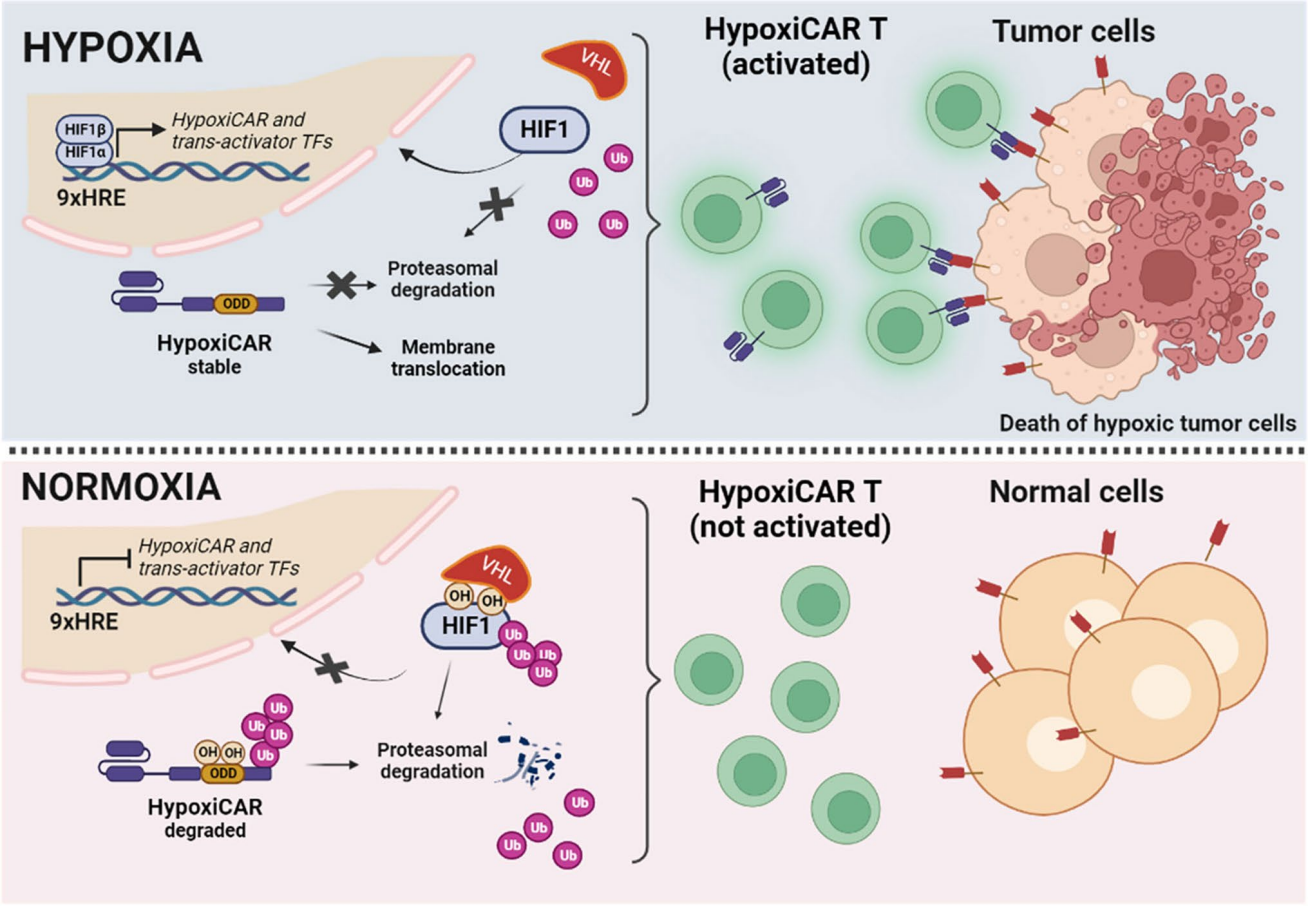
Mechanisms of hypoxia-sensitive CAR T activation. Representation of the selective activation of the hypoxia-sensitive CAR T cells able to better discriminate tumor cells from healthy cells by exploiting the hypoxia of  the tumor microenvironment. The coding sequence of the CAR has been engineered, introducing nine repeats of HRE in the promoter and an ODD sequence in the C-end of the CAR. Thus, the expression of the hypoxiCAR is enhanced at low oxygen pressure being under the control of HIF-1α/HIF-1β (upper panel). In addition, the presence of the ODD domain guarantees a post-translational control on the hypoxiCAR stability, being degraded at normal oxygen pressure (low panel) or stabilized and exposed on the plasma membrane in hypoxic conditions (upper panel). The figure was created with Biorender.com

**Fig. 5:**
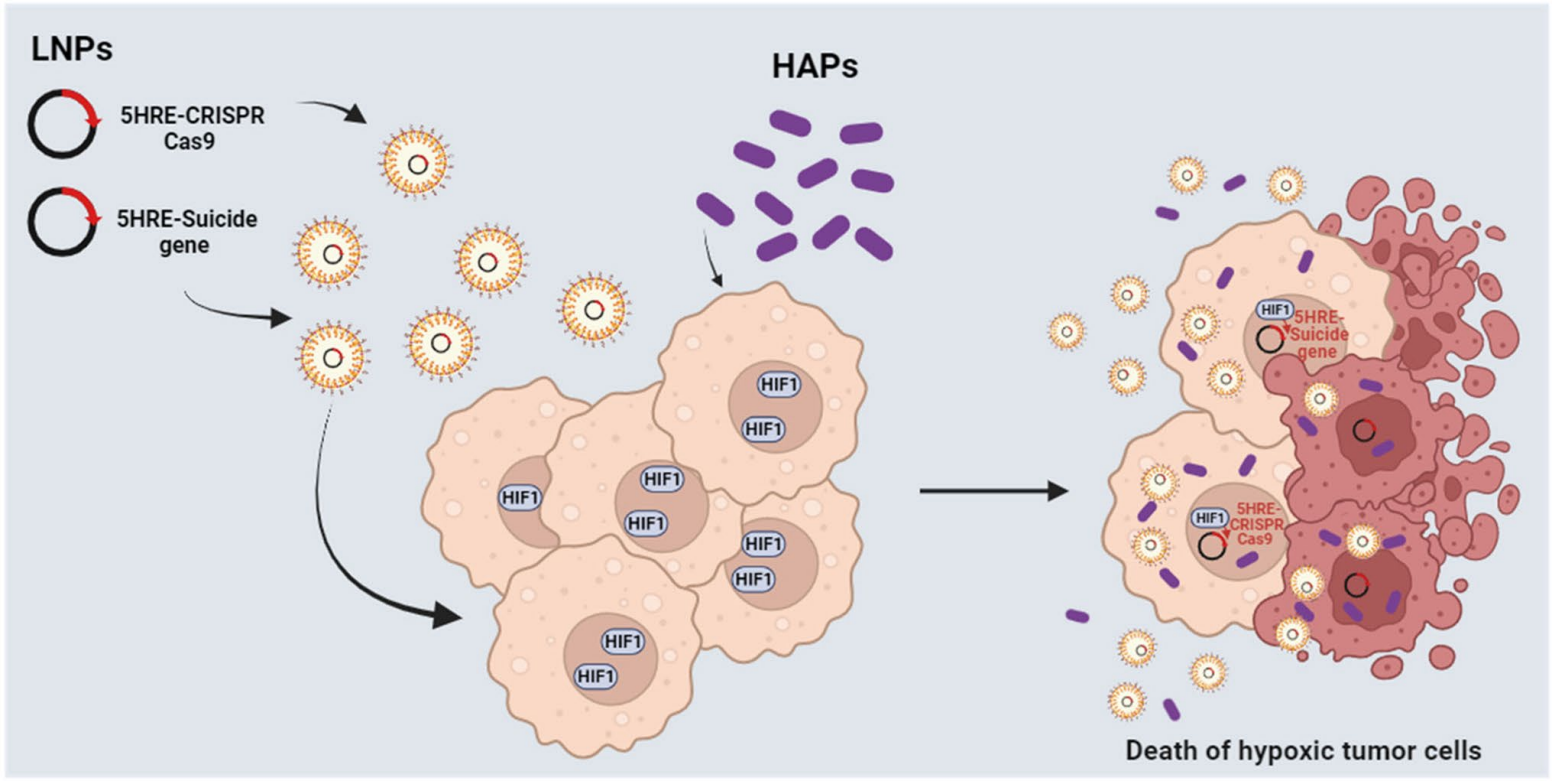
Alternative strategies to target hypoxic tumors. Schematic representation of additional strategies under implementation to target hypoxic tumors. LNPs, lipid nanoparticles encapsulating plasmids for the expression of suicide genes or CRISPR-Cas9 under the control of the VEGF promoter (5xHRE). HAPs, hypoxia activated prodrugs. The figure was created with Biorender.com

The first strategy is related to the improvement of the safety of CAR T. In most CAR T systems, the recognition of tumor cells depends on the bio-distribution of the CAR. Thus, on-target/off-tumor toxicity can occur due to the presence of the antigen also on non tumoral cells, as in the case of anti-ErbB2 CAR T cells for the treatment of metastatic colorectal cancer. Accordingly, several strategies have been experimented to spatiotemporally control the activity of CAR-T, including the tumor hypoxic microenvironment. Particularly, one approach was based on the idea of creating an oxygen-sensitive chimeric antigen receptor by fusing the CAR with the ODD HIF domain (ODD-CAR), in order to create a hypoxia-dependent molecular “switch” of the exposure of the ODD-CAR on the membrane of the engineered T cell. Basically, in normoxia/physoxia the ODD-CAR would be polyubiquitinated and degraded. Conversely, in tumor hypoxic sites, the ODD-CAR would be stabilized and consequently exposed on cell surface, allowing a more selective antigen recognition by CAR T [213]. However, a low level of off-tumor killing activity was still observed. An upgrade of this system was recently proposed by Kosti and colleagues, which increased the stringency of hypoxic tumor cell recognition through the insertion of a dual oxygen-sensing control element. Beyond the fusion of the ODD sequence at the C-terminus of the CAR, a 9-tandem repetition of HRE was introduced in the promoter of the CAR, creating what the authors called the “HypoxiCAR” (HiCAR) (Fig. 4) [214]. The promising efficacy of this approach has been proven in vitro and in vivo, overcoming two main limitations, i.e., a more stringent control of CAR expression and a greater anti-tumor activity with no apparent on-target/off-tumor toxicity. In addition, a gene panel including *VEGFA, PGK1*, *CA9*, *SLC2A1,* and *ALDOA* has been proposed to select those patients who could best benefit from this promising approach [215]. He et al*.* proposed an additional strategy to significantly increase the expression of the CAR in hypoxia compared to normoxia/physoxia, by transforming the trans-activator system Gal4-UAS (upstream activating sequence) into an oxygen-dependent one [216, 217]. More specifically, the authors integrated the HiCAR system with a conditional trans-activator protein made of a zinc finger portion (ZFD, zinc finger domain), that specifically recognize the UAS (ZFDBD, zinc finger domain-binding domain), fused with an ODD domain for oxygen-dependent regulation. In addition, multiple HRE sequences have been introduced in the promoter region of the trans-activator, creating a bidirectional control exerted by HIF, both on the expression of the CAR and on the trans-activator, creating what the authors called “HiTA-CAR T”, which is a bidirectional hypoxia-inducible transcriptional amplification system [218]. This allowed a strong and a more stringent induction of CAR expression under hypoxia, not observed in normoxia, with not reported off-tumor toxicity, thus improving the safety of the CAR T [216]. However, beyond ErbB and Her2, more CAR antigens have to be tested and more preclinical tests have to be performed to guarantee the safety of the developed systems [219, 220]. To be noted, these strategies remind those employed to synthesize prodrugs targeting hypoxic tumor tissues, according to which the prodrug is activated by hypoxia (HAPs, hypoxia-activated prodrugs) (Fig. 5) [221]. Unlike HiCAR T or HiTA CAR T, several HAPs entered clinical trials, even in combination with radio- and chemotherapy [222]. However, none of them has been approved for clinical therapy and needs further investigation, probably due to a lack of patient stratification in relation to hypoxia tumor level [223].

The second very recent strategy that exploits tumor hypoxia for cancer therapy is related to the so called suicide gene therapy. This strategy represented a clear step forward for gene therapy, finding appealing applications for cancer therapies as well, since it can drastically reduce the toxic effect on healthy cells [224, 225]. Particularly, this system contemplates the delivery (with viral or non-viral vectors) of a transgene (viral or bacterial) into tumor cells in order to activate a prodrug or to express the “suicide” gene product in the targeted tumor cells (Fig. 5) [226]. However, this strategy is not completely free from toxic effects. Indeed, a “bystander effect” has been observed in adjacent cells of several treated tumors [227]. An emblematic example of this strategy is the suicide HSV-tk/GCV system, in which the thymidine kinase gene of *Herpes simplex virus* (HSV-tk) phosphorylates ganciclovir (GCV), a synthetic analog of 2′-deoxyguanosine, to GCV monophosphate, that can be converted by cells into the triphosphate form. This latter is incorporated into DNA, determining mainly a delay of proliferation and consequent apoptosis of tumor cells [228]. Some authors investigated the possibility of exploiting this suicide gene system to target hypoxic cells by putting it under the control of HIF. Interestingly, the CRISPR-Cas9 system has been experimented for cancer therapy, due to its ability to disrupt the expression of undruggable driver genes or oncogenes, but not in hypoxic condition. Last year, Davis and colleagues proposed and validated a technology that coupled HRE-driven HSV-tk and CRISPR-Cas9 delivered in lipid nanoparticles to target hypoxic tumor cells and induce their cell death. More specifically, the authors used CRISPR-HRE-Cas9 to abrogate the expression of genes involved in multidrug resistance and the HIF-guided HSV-tk to potentiate the killing effect [229]. Although this technology needs improvement, the choice to combine CRISPR-Cas9 with suicide gene therapy seems promising. Therefore, deepening the knowledge related to the alterations of hypoxia-response pathway in cancer cells and to tumor-specific molecular mechanisms could undoubtedly help to improve both HIF targeting and gene editing strategies, and finally the stratification of patients that can benefit from such treatments.

## Conclusion and future perspectives

The astonishing genome editing development  marks the fulfillment of the human hope to eradicate hereditary or acquired human pathologies, including cancer. Although certainly associated with important bioethical and regulatory problems, the techniques developed are important to open up scenarios unimaginable until now.

Numerous methodological features need to be improved, some of which substantially affect the possibility of editing the human genome and have been discussed here, e.g., the effectiveness and precision of delivery, the percentage of targeted cells, and the reduction of off-targets. Furthermore, while for some genetic diseases it does not appear necessary that all cells are targeted, the percentage of edited cells appears to be of fundamental importance for diseases such as cancer.

The molecular system of metabolic and functional adaptation to O_2_ pressure variation appears to be fundamental to the survival of individual cells, tissues, and the whole organism. The genome editing of hypoxia-related genes can have two major objectives. The first is to handle the hypoxia-responsive mechanisms hampering the survival of targeted cells (i.e., cancer cells) at low O_2_ pressures. This objective appears important for exploiting the hypoxic condition of solid tumors, especially in the stem cell component, and may be partially achievable. The second objective is the correction of mutations present in some specific tumors. Indeed, as described here, mutations of VHL, HIF, and PHD were demonstrated in several cancers. This second objective is theoretically achievable based on BE and prime editing developed so far. On the other hand, the number of genetic mutations, including those related to the response system to O_2_ variations, is too heterogeneous to be considered achievable. In the near future, true genetic precision medicine will be a reality.

## Data Availability

Not applicable.
